# Maintaining animal-source food production in conflict zones: lessons from Ukraine

**DOI:** 10.1186/s13028-025-00850-5

**Published:** 2026-01-07

**Authors:** Natalia Mammadova, Pavlo Levchenko, Hedvig Gröndal, Susanna Sternberg Lewerin, Elisabeth Rajala

**Affiliations:** Department of Animal Biosciences, Swedish University of Agricultural Sciences, PO Box 7023, 750 07 Uppsala, Sweden

**Keywords:** Animal production, Animal-sourced food, Food security, Livestock, Preparedness, Resilience, War

## Abstract

**Background:**

The deteriorating security situation in Europe’s immediate neighbourhood has underscored the importance of safeguarding food systems during conflict. Animal-sourced foods are essential for human nutrition and play a critical role in maintaining national resilience, yet their production becomes highly vulnerable in wartime. This study explores the resilience of Ukrainian animal-sourced food systems following the 2022 Russian invasion, focusing on the perspectives of farmers and veterinarians.

**Results:**

Through 18 in-depth interviews with farmers and veterinarians across occupied and non-occupied regions, the study examines perceived challenges, adaptive strategies, and preparedness levels. Respondents reported severe disruptions, especially in occupied areas, including breakdowns in feed supply chains, delivery of medicines and other essential logistics, prolonged power cuts, reduced livestock production, livestock losses, and staff shortages. Adaptation strategies ranged from diversification to increased self-sufficiency, though outcomes varied widely. The absence of crisis preparedness plans led to improvised responses in the early stages of the conflict. Interviewees highlighted key factors for strengthening livestock and food system resilience during crises, emphasizing human resources, technical preparedness, and contingency planning.

**Conclusions:**

The findings of this study highlight the importance of preparatory planning, resource reserves, skilled personnel, and support networks. The experiences of Ukrainian farmers and veterinarians provide important insights into how agricultural systems can become more adaptive and responsive during future crises, emphasizing the need for flexibility, preparedness, and community collaboration. However, further research encompassing a wider geographic scope and a broader range of stakeholders is needed to validate these findings.

**Supplementary Information:**

The online version contains supplementary material available at 10.1186/s13028-025-00850-5.

## Background

The global security landscape is marked by instability and unpredictability, with a significant deterioration in the security situation across Europe’s immediate neighbourhood [1]. In response, the European Parliament has emphasized the need to increase agricultural resilience to external shocks and reduce Europe’s dependency on imports of critical inputs such as fertilisers and plant-based proteins for animal feed [2]. A key aspect of crisis preparedness is strengthening society’s ability to prevent and respond to crises while ensuring the continued functionality of essential services, including food production and supply. Ensuring access to safe drinking water and food is a fundamental component of national security, necessitating coordinated efforts across individuals, businesses, municipalities, and government agencies [3].

Animal-sourced foods play a critical role in human nutrition, providing essential macro- and micronutrients [4]. However, they also serve as potential carriers of zoonotic diseases, posing risks to public health. Consequently, a resilient and sustainable livestock production with healthy livestock is vital for maintaining food security and safety [5]. The importance of resilience in European farming systems has been increasingly recognized in agricultural policy, with recent studies highlighting significant regional and farm-type variability in robustness, adaptability, and transformative capacity [5]. For instance, during the COVID-19 pandemic, farmers across Europe had to rapidly adapt to supply chain disruptions, as exemplified by Austrian farmers who shifted to direct marketing via online platforms when traditional food markets closed in 2020 [6].

Despite the recognized importance of food system resilience, there is a notable lack of scientific data on how farmers and veterinarians, who among others are key actors in the production of animal-sourced foods, are affected in conflict zones. Existing studies provide some insights: for example, research in Nigeria published in 2013 found that a majority of surveyed farmers lost productive land due to conflict, leading to declines in sheep and goat meat production [7]. Similarly, a much older study from what was then Rhodesia (now Zimbabwe) documented how the disruption of veterinary services during a 7-year conflict led to severe outbreaks of zoonotic and livestock diseases, including foot-and-mouth disease, anthrax, and rabies, resulting in significant human and animal fatalities [8]. A slightly more recent study in Afghanistan reported a marked increase in livestock mortality in conflict-affected regions, largely due to the suspension of vaccination programs and anthelmintic treatments [9]. Reports of high rabies incidence but low numbers of submitted samples in Ukraine demonstrate the challenges linked to maintaining surveillance and control of serious diseases in times of armed conflict [10]. These findings underscore the need to build robust preparedness measures in peacetime to mitigate the impacts of crises and armed conflicts.

The ongoing war in Ukraine provides a contemporary case study of how armed conflict disrupts food production systems. One year after Russia’s invasion unprecedented consequences for global agricultural markets, food security, and nutrition were reported [11]. Figures from 2022, show that attacks on Ukraine’s agricultural infrastructure resulted in the destruction or damage of over 84,200 pieces of agricultural machinery, the loss or theft of four million tons of grains and oilseeds, and significant damage to storage facilities for 9.4 million tons of agricultural products [11]. Additionally, the war severely impacted Ukraine’s energy infrastructure, with over 30% of the country’s power grid damaged by Russian strikes as of December 2022 [12]. This has led to major disruptions in the dairy sector, as processing plants face difficulties operating under emergency power shutdowns, thereby affecting both food supply and price stability.

The overall aim of this study was to analyse Ukrainian farmers’ and veterinarians’ perspectives on the resilience of animal-sourced food production systems during the armed conflict in Ukraine with special focus on challenges and lessons learned. The study had two specific objectives:

i. to explore farmers’ and veterinarians’ perceptions of the challenges faced and adaptive strategies adopted by farmers, veterinarians, and other key stakeholders to sustain food production during wartime; and
ii. to explore farmers’ and veterinarians’ experiences of the level of preparedness among these groups prior to the conflict. Insights gained from this research may contribute to shaping more effective crisis-response strategies and food security policies in other European countries.

## Methods

The study draws on qualitative methods and in-depth interviews with key Ukrainian stakeholders in livestock production: livestock farmers and farm managers—spanning the dairy, beef, pork, and poultry sector; livestock veterinarians from various regions of the country and one representative from the dairy industry. Qualitative studies are widely used within the social and medical sciences and are increasingly recognized within veterinary science as well [13]. We aimed for variation in animal species, production form, farm size and geographical location (occupied and non-occupied areas) since we hypothesized that these factors could potentially be linked to different experiences [14]. Our intention was to interview as many informants as possible, but with no numerical target. The participants were identified via personal contact networks, via social media platforms, email or telephone.

Two different interview guides were developed, one for the veterinarians and one for the farmers and farm managers, with approximately 7 questions each (Additional file 1). The interview guides were pre-tested with one farmer and one veterinarian to allow for improvements and included both open-ended and closed questions. The questions for farmers and farm managers covered aspects such as farm characteristics, challenges connected to livestock production, adjustments in the production that have been necessary, key factors seen as essential for securing food production, and the level of preparedness in place on the farm before the war.

The questions for veterinarians covered challenges and adjustments connected to their daily work, their level of preparedness in place before the war, and key factors that they see as essential for securing animal food production during a crisis or conflict. All participants were also encouraged to reflect on related matters that they found relevant.

All individual in-depth interviews were conducted orally between June and September 2024 via the digital platform Zoom (Zoom Communications, Inc, San José, California, USA) by two of the authors (PL and NM). The interviews were carried out in either Ukrainian or Russian, depending on the participants’ native language. All interviews were recorded, transcribed, and translated into Swedish by NM. Thematic analysis, an approach commonly used in qualitative research, was conducted to identify and interpret patterns and themes within the interview data [15, 16]. In parallel with the manual translation process, an initial analysis was conducted and descriptive themes inspired by the question guide and informed by relevant literature, were developed. In addition, the recordings were transcribed and translated by an AI tool (Transcribe, Routes Software SRL, Lomazzo, Italy) to help identify quotes across all interviews illustrating the themes. These AI-generated excerpts were then double-checked and verified by NM and PL and read by ER and HG. The themes where subsequently adjusted based on discussions in the research group. The thematic analysis primarily followed an inductive approach, allowing themes to emerge directly from the participants’ narratives rather than being imposed by pre-existing theoretical frameworks.

### Result

### Participants

A total of 18 respondents participated in the study, including 14 men and 4 women (Table 1). The interviews ranged in length from 16 to 70 min. Three respondents were farm managers, eight were farmers, one worked as a technician in a dairy company, and six were veterinarians (one of these represented a state veterinary clinic and the others were employed on farms). The respondents came from various regions across Ukraine, including four regions that had been occupied by Russian forces. Eight of the farms had been occupied for various lengths of time, but none of the farms were occupied during the interview. Seven farms were engaged in dairy production, with a large variation in herd sizes—ranging from 200 to 2200 dairy cows, and four of the farms were also involved in beef production. Pig production was present on seven farms, ranging from small-scale producers with just a few sows to large-scale operations with thousands of sows. Three farmers focused on poultry production: one operated a small-scale subsistence farm, while the other two ran large-scale commercial farms. One farm was completely destroyed during the war, and another was severely damaged. A farm located in eastern Ukraine was evacuated to the western part of the country in the beginning of 2022.

**Table 1:** Characteristics of study participants (*n* = 18)

No.	Role	Gender	Production	Occupied	Farm size
1	Farmer	Man	Dairy	Yes	Large scale
2	Farmer	Woman	Dairy	No	Large scale
3	Farmer	Man	Dairy, beef	No	Large scale
4	Farmer	Man	Pig	No	Small scale
5	Farmer	Man	Pig	Yes	Medium scale
6	Farmer	Man	Poultry	No	Large scale
7	Farmer	Man	Pig	Yes	Medium scale
8	Farmer	Man	Poultry and pig	No	Large scale
9	Farm manager	Man	Pig	No	Large scale
10	Farm manager	Woman	Pig	No	Large scale
11	Farm manager	Man	Poultry	Yes	Large scale
12	Veterinarian	Woman	Pig	No	Medium scale
13	Veterinarian	Man	Dairy, beef	Yes	Large scale
14	Veterinarian	Man	Dairy	Yes	Medium scale
15	Veterinarian	Man	Dairy, beef	Yes	Large scale
16	Veterinarian	Man	Dairy, beef	No	Large scale
17	State veterinarian	Woman	Various livestock	No	Small to large scale
18	Technician	Man	Dairy company	Yes	Large scale

The analysis of the interviews identified seven themes describing the experience and lessons learned in maintaining animal-source food production in Ukraine following the Russian invasion: Drivers and consequences of production changes; Electricity, fuel and access to clean water; Feed supply and logistics; Veterinary services, animal health, and biosecurity; Workforce challenges; Preparedness plans, and state support after the invasion; and Key factors for farm resilience.

### Drivers and consequences of production changes after the Russian invasion

While all interviewees described that the Russian invasion had required large adjustments, their experiences varied depending on if they worked in farms within or outside areas occupied by the Russian troops. Table 2 summarises key findings comparing farms in occupied and non-occupied areas connected to drivers and consequences of production changes after the Russian invasion, and Table 3 presents illustrative quotes connected to this theme.

**Table 2:** Drivers and consequences of production changes across farms in occupied and non-occupied Ukraine

Farms in occupied areas	Farms in non-occupied areas
Production losses and operational disruptions • Loss of livestock and significant declines in production resulting from factors such as feed shortages, and reduced milking frequency • Closure of slaughterhouses and disrupted logistics, causing milk disposal, overcrowding of animals, or forced redistribution of products	Production adjustments • Deliberate production reductions (to protect animal health) • Stable production where herds and inputs were maintained
Market access and logistics • Cut-off from dairy processors and feed suppliers • Feed shortages leading to emergency measures such as donating animals to civilians, and releasing livestock which led to severe animal welfare issues	Market access and strategy shifts • Farms diversified into value-added processing • Some producers shifted market channels (e.g., from wholesale to processed products) • Farms generally maintained access to feed and processors, though with delays. • Panic buying at the start of the war sometimes increased demand, especially for eggs and poultry

**Table 3:** Illustrative quotes on drivers and impacts of Ukrainian livestock production changes after the invasion

All work was kept to a minimum, you only tried to keep it running. […] Nothing extra was done to improve the conditions. Only the most basic was done to keep the animals alive (Interview no 1) […] the biggest problem that arises at the beginning of a situation like this is the production stoppage. The dairies did not accept milk. And I have already said that we have 70–80 tons of milk per day, and we must get rid of it somehow. (Interview no 2) And when the war began, well, we converted our entire farm in, I would say, about two months. We were already producing canned goods, pâtés, and partially sausages. Then we started, yes, it didn’t last very long, but we started a line for pies and dumplings. But then we hired people, because it was very difficult to do everything ourselves. They started feeding people at the roadblocks. Then they delivered food to the [military] units. There were many units gathered there at that time. We cooked food. We had arranged a dining hall. (Interview no 4) [About sows being let out] About 100 sows. And on each one, about 10–12 piglets. Something like that. Yes, where they went, ran off to, I don’t know, I can’t say. Terrible. (Interview no 5) I explained that we could not and did not have the moral right to simply abandon everything and leave. (Interview no 14) [Regarding the reduction from three to two milkings.] And the animals, well, at first they were anxious, they were used to being milked. But then, as there was no concentrated feed, their production gradually decreased. (Interview no 14) [….] We didn’t reduce the herd at all. For the people, this is their job, and […] it’s food security for people. (Interview no 3) [About discarding milk] Then we started pouring it out on… Well, on the road, on the asphalt. Then I decided to collect it anyway and use it to feed the calves, the young animals. (Interview no 14) [About requirements for meeting EU standards]. I have to say that when we first saw the requirements we had to meet, many of my colleagues simply gave up and shut down their operations. Not all of farming, but the milk production. We started to improve, even though it was difficult, and step by step we reached the level we are at today. And we meet all those standards. (Interview no 13) […] We didn’t hold back from anyone, there were queues 50 m long to get milk. So we just distributed milk. (Interview no 14) [About overcrowded stables]. Come and get your heifers, we have so many of our own. We […] had many of our own heifers, and theirs were taking up space. We understood them when they said: We can’t come get them, there are tanks here, the military won’t let us. Somehow we arranged space in the calving pens and converted areas so that the heifers could calve there. (Interview no 13)	

Among the respondents, four farmers, three veterinarians, and one employee at a dairy facility were working on holdings in areas that had been occupied. All these eight interviewees described major adjustments triggered by the Russian invasion. Several recurring topics emerged: declining production, severe disruptions in logistics, the loss of livestock and personnel, and a strategic reorientation towards self-sufficiency and diversification, exemplified by initiatives such as on-farm food production and various forms of value addition such as butchering or even producing ready-to eat food products. There was also a pronounced emphasis on safeguarding the well-being of personnel and ensuring the survival of livestock, rather than focusing solely on financial returns.

Four of the interviewees working on farms with dairy production found themselves operating in occupied territory. They were forced to reduce milking frequency from three to two times per day, resulting in a sharp decline in milk yields. One reported that daily production decreased from approximately 25–35 L per cow before the occupation to just 10–15 L thereafter. Contributing factors included limited access to concentrated feed, heightened stress levels among the animals, and essential staff mobilized to the front or leaving their positions due to safety concerns. In the early phase of occupation, farmers were cut off from dairy processors, forcing them to either distribute milk to local communities, repurpose it as calf feed, or discard it entirely. The shutdown of slaughterhouses also created significant challenges, leading to overcrowding in some farms. Over time, however, some operations managed to resume sales by establishing alternative logistics channels and accessing new markets. One farmer specifically mentioned the European Union and Asia as key destinations.

Two interviewees involved in pig farming also found themselves in occupied areas, suffering both direct destruction and acute logistical breakdowns. One interviewee recounted that during the 2014 invasion of Crimea, nearly their entire herd was lost and equipment destroyed. Although they managed to partially restore operations, the 2022 invasion once again brought production to a complete standstill. Both interviewees connected to pig production reported severe shortages of feed and water, leading them to either donate pigs to the civilian population or release them into the areas surrounding the farm. The latter caused animal welfare issues that were described as horrific—for instance, the carcasses of dead pigs emitted odours that attracted feral dogs, which subsequently hunted the surviving animals. Reconstruction efforts have since commenced on one of the farms following liberation, but operational capacity remains severely constrained.

One of the poultry producers ended up in an occupied area and faced devastating impacts, including the loss of hundreds of thousands of birds. Despite this, the producer chose to resume operations at a new location and has since succeeded in more than doubling their production. At the time of the interview, preparations were also underway to expand into the processing of poultry meat, demonstrating a resilient and forward-looking approach despite the setbacks encountered.

The interviewees operating outside of Russian-occupied territories reported varying degrees of production changes in response to the war, largely influenced by market disruptions, logistical constraints, and a focus on sustaining animal welfare and workforce stability.

Among dairy producers, responses varied. One producer deliberately reduced milk output to prioritise animal health, adjusting feeding regimes to less concentrated diets, which led to a temporary drop in production and animal weight loss. In contrast, another dairy and meat producer maintained stable production levels, opting not to reduce herd size or change production strategies to avoid long-term losses in productivity. Another integrated producer of milk, meat, and grain also maintained production volumes but faced initial losses due to market instability, with disruptions in milk collection causing early uncertainty. Nevertheless, they later stabilized operations and resumed exports.

Pig farming enterprises outside of occupied territory exhibited a range of adaptive strategies. Some producers made no significant changes to production volumes but introduced genetic improvements, such as crossbreeding for enhanced meat yield, and renewed boar stocks. Others shifted their market orientation, transitioning from wholesale to value-adding pork processing, including the production of preserved goods like sausages and pâté, to secure higher margins and meet domestic demand. Another pig farm, benefiting from population movements from occupied areas, and military demand, expanded sow numbers from 400 to 700 and increased the total herd to several thousands. They also transitioned to higher-yield breeds and switched to commercial premixed feed to ensure quality and consistency. Another pig producer emphasized the need for rapid processing and sales to mitigate market volatility, accelerating production cycles and engaging administrative staff in farm operations to address labour shortages. Despite challenges, maintaining workforce retention and timely salary payments remained a priority.

The poultry farms outside of occupied areas largely maintained stable production levels, though strategic adjustments were made. One producer shifted focus from broiler meat to egg production due to its lower market risk and easier sales during wartime. To safeguard livestock, they relocated much of their production to western Ukraine, where security conditions were relatively better. Increased consumer demand, driven by panic buying in the beginning of the war, further influenced production decisions. One farm described that the war led to an enormous increase in the demand for eggs and birds ”*since people started to panic and bought everything so to speak*,* in order to stock up*” (Interview no 6). Another poultry enterprise reported stable operations and continued feed production, although they experienced a 10–15% reduction in workforce due to military mobilization.

### Challenges with electricity, fuel and access to clean water

All interviewees, whether located inside or outside occupied areas, described frequent and prolonged power outages as a major challenge for their operations. These disruptions not only hindered daily activities but also drove up operational costs and created long-term uncertainties about production continuity. Damage to the electrical grid, deliberate destruction of infrastructure, and proximity to frontlines led to extended periods without electricity—in some cases lasting over a month.

The power cuts severely affected critical farm functions such as feed preparation, milking systems, water supply, ventilation, and refrigeration. Many farms acquired generators to sustain essential operations; however, these systems were often insufficient or suffered mechanical failures under prolonged use. Furthermore, the rising demand for electricity—particularly during extreme weather events such as the summer heatwave of 2024—placed additional strain on already limited resources, as described by one farmer. Also, the situation was exacerbated by fuel shortages becoming a critical bottleneck. With diesel and gasoline supplies highly restricted, due to skyrocketing prices and, in some areas, occupation forces seizing control of fuel depots, farmers were forced to ration fuel strictly. Even farms with initial reserves soon faced critical shortages, prioritizing essential activities such as running generators, maintaining water supply, and transporting milk to dairies. Some resorted to borrowing or purchasing fuel from neighbouring communities to keep their operations running.

Access to clean water was closely linked to the availability of electricity and fuel. While many farms had their own wells, reliance on electric pumps made water supply vulnerable to power cuts. If generators were available and functioning, water access could be maintained. However, fuel shortages and mechanical failures of generators frequently jeopardized water availability, complicating animal care and farm operations—especially during freezing winter temperatures, when the lack of electricity led to frozen water pipes. Bombing of water infrastructure in some regions further exacerbated the situation. Some farms near conflict zones reported that attacks on local pump stations forced them to seek alternative water sources, including transporting water from nearby lakes or collecting rainwater, although these measures were risky and unreliable due to ongoing hostilities.

Furthermore, the combination of electricity shortages and limited fuel and water supplies led to secondary losses, including equipment failures, fires caused by overheating machinery, and the spoilage of stored products. Despite proactive efforts to stockpile fuel and secure backup power sources, many producers struggled to maintain stable production during these major stresses. See Table 4 for illustrative quotes.

**Table 4:** Illustrative quotes from Ukrainian farmers and veterinarians on electricity, fuel, and water access

[…] for the sowing campaign, which was due to start in March, we had already stocked up on fuel. This fuel was bought in advance because it came in one big delivery. It saved us. Nobody thought—and everyone assured us that there would be no war, that it was just a game. (Interview no 3) If we could go back in time, we would have bought that generator earlier. It would have solved our situation for a couple of months. (Interview no 12) Yes, when all of this started, when they started shooting at the power supply, we bought two of them [generators] in the beginning, during the first days. We paid too much money for them, because there was such hysteria around these generators. (Interview no 13) We didn’t have any problems with water because we had wells—yes, each farm has its own well. (Interview no 10) […] people even tried to fetch water from a nearby lake. (Interview no 7)	

### Challenges with feed supply and logistics

The interviewees described how the conflict caused profound disruptions to both feed supply chains and other logistics, with destruction of roads and bridges and in some cases entire regions subjected to hostile control. The combined impact of these shortages and infrastructural failures severely undermined farm operations, resulting in substantial economic losses and, in extreme cases, making it necessary to either abandon livestock or slaughter entire herds or flocks. A majority of respondents (12 out of 18) reported major difficulties with feed procurement, largely due to the destruction of infrastructure and widespread logistical breakdowns. Only a few farms with self-sufficient feed production stated that they had avoided these challenges. Shortages of critical feed components, particularly soybean meal—a primary protein source—forced many farms to adjust their feeding strategies, substituting soy with lower-quality alternatives such as sunflower meal and corn feed. One dairy producer reported a reduced milk yield by approximately 2.5–3 L per cow, poorer animal health, and extended gestation periods by an estimated 4–5 days.

Power cuts further exacerbated the situation, halting production at farms with on-site feed processing facilities. Although some farms had reserves of concentrate or grain, transport bottlenecks severely limited access to both external supplies and existing stocks. One producer, whose facility was in the middle of a conflict zone, experienced catastrophic losses. Despite holding substantial maize reserves (~ 3000 tonnes), the farm was forced to slaughter all adult poultry and distribute them free of charge to the local population due to an inability to access and process these feed stocks, resulting in significant economic losses. To supplement limited resources, another farm increasingly relied on roughage to conserve concentrate reserves, but these measures proved insufficient under prolonged crisis conditions.

Blocked transport routes not only restricted access to feed and fuel, but also prevented the movement of live animals and products to markets. In some areas, logistics systems ceased functioning entirely, forcing farms to delay or halt production. One farmer reported that export contracts were cancelled, forcing them to sell meat and dairy products on the domestic market at significantly lower prices. Even after regions were de-occupied, the extensive damage to transport infrastructure continued to impede the recovery of supply chains on many farms. See Table 5 for illustrative quotes.

**Table 5:** Illustrative quotes by Ukrainian farmers and veterinarians connected to challenges with feed supply and logistics

[About a high-producing cow] Stopping it is like trying to stop a Mercedes on the Autobahn. Do you understand? It’s very difficult. […] And of course, because of the feed, due to the introduction of large amounts of straw, milk production started to decline. (Interview no 2)	
Logistics, that was a difficult issue—especially during the first 2 months, it was very hard to find anything at all, no one understood what was happening, where to go, or what to do. (Interview no 12)	

### Challenges in veterinary services, animal health, and biosecurity

A vast majority of the respondents reported that the conflict severely disrupted access to veterinary services, medicines, and sometimes also semen and artificial insemination (AI) services. Although many of the farms initially had stockpiles of medicines and vaccines, supply chain disruptions and staff shortages quickly led to critical shortages, particularly of vaccines, disinfectants, and AI supplies. The mobilization of veterinary personnel and the departure of specialists further strained the availability of services. Many farms experienced delays in preventive healthcare, delaying animal vaccination for weeks. Stress from constant shelling led to animal injuries and poor animal welfare. While the situation gradually improved over time on most farms, with external assistance and the re-establishment of vaccination routines, the early months of the conflict were marked by significant challenges in animal health management and disease prevention at many farms.

Three respondents reported an increased incidence of animal diseases, one pig farm, and two dairy farms. A dairy farm with approximately 500 animals experienced ten cases of pneumonia among young animals, the other dairy farm saw an increase in cases of acidosis/ketosis due to imposed changes in feeding and milking routines. The pig farm reported that the introduction of new animals led to outbreaks of infections such as mycoplasma and other diseases, contributing to increased workload and stress. Furthermore, emergence of new diseases was associated with herd expansion. Additionally, two pig farms reported signs of African swine fever, but no confirmed cases occurred on either farm. However, outbreaks of African swine fever were reported in the surrounding area.

At one large dairy farm, bombings resulted in the loss of 27 animals, with many others sustaining severe injuries, including shrapnel wounds and deep lacerations. One large cattle farm reported the loss of 35 animals due to various injuries. Another pig farm reported that biosecurity measures were compromised due to ongoing construction and staff shortages, affecting the maintenance of safety protocols. The veterinary clinic reported an increase in rabies cases, attributed to the suspension of wildlife vaccination programs in 2022 due to the onset of the war. In response, door-to-door preventive rabies vaccinations were initiated. Also, one dairy farm reported a decreased incidence of mastitis, which they attributed to reduced productivity. See Table 6 for illustrative quotes.

**Table 6:** Illustrative quotes by Ukrainian farmers connected to experiences with veterinary services, animal health, and biosecurity

[about African swine fever] How we struggled, we completely isolated ourselves. […] Started taking care of biosecurity more, more actively—how should I put it—more seriously, you could say. (Interview no 4)	
[…] all our vaccines are foreign. At the beginning of the war, there were problems with everything. (Interview no 3)	
When productivity fell, the number of new cases of mastitis in the cows also decreased significantly. There were almost none. And if there were any, I can say that no one cared particularly much about it. (Interview no 13)	

One of the interviewed veterinarians, working across multiple farms, reported significant organizational restructuring, including the consolidation of districts that reduced the geographical area of responsibility. While these changes streamlined certain aspects of the work, they also introduced new challenges to veterinary practice and service delivery.

### Workforce challenges

Most participants reported that the war led to significant workforce disruptions, with many employees mobilized to the front or leaving their positions due to safety concerns, or even being killed when the farm was bombed. This resulted in acute labour shortages and increased workloads for the remaining staff, many of whom had to take on multiple roles and responsibilities. Women, including those nearing retirement age, increasingly performed physically demanding tasks traditionally handled by men. Despite efforts such as wage increases and rapid training of new recruits at some farms, the loss of male workers, in particular, has been difficult to compensate for. While some farms reported only marginal staffing changes, others faced catastrophic shortages, with up to 80% of workers evacuating during periods of active conflict. Over time, staffing levels have somewhat stabilized, but recruitment remains challenging at many farms, especially of male workers. Some respondents reported significant psychological strain among employees, including cases of fatalities and displacement, yet they also demonstrated a strong resolve to rebuild and adapt to the harsh realities of war. See Table 7 for illustrative quotes.

**Table 7:** Illustrative quotes by Ukrainian farmers and veterinarians connected to workforce challenges

We’re keeping them in this situation with a fairly high salary because we’ve reviewed the salary; we’re keeping people so that they don’t move, and some people we’re just reserving, since it’s critical infrastructure, the food industry, so it’s allowed to reserve, and some people are just reserved. […] When it comes to specialists, it’s not possible to find a specialist. (Interview no 3) Yes, first and foremost it affects the staff resources. I see that there’s no one who can work, and that’s the scariest thing. You can buy tractors, but not people… (Interview no 14) Our biggest problem at present, just like since February 24th, is the staff. First, some fled, then many were mobilized, and the mobilization is still ongoing. Our main issue is the staff and who is going to work. (Interview no 15)	

### Preparedness plans before 2022 and state support after the invasion

All interviewees reported that they did not really believe there would be a war and were therefore unprepared. Most described limited preparedness, with no formal crisis plans in place. Farms typically maintained basic reserves of feed, fuel, water, and medicines, sufficient for only 1 to 2 months, which proved inadequate for prolonged disruptions. A few farms had generators to secure electricity, but energy supply remained a vulnerability, and most lacked dedicated shelters or clear evacuation plans for livestock. Overall, preparedness relied on basic self-sufficiency rather than structured crisis management. Although there was some awareness of potential risks, the prospect of full-scale conflict seemed remote, and contingency planning was minimal. When the invasion began, the interviewed farmers and farm managers were severely affected by supply chain disruptions and shortages. With limited access to state aid, many farms relied on external support and improvised solutions, such as acquiring additional generators and stockpiling essential resources, to sustain operations. A few respondents noted that government support programmes were suspended or difficult to access during the war, leading to low expectations for state assistance. Instead, neighbours and personal networks emerged as vital sources of support. These experiences have since driven some farms to strengthen their resilience and develop more robust emergency preparedness strategies. See Table 8 for illustrative quotes.

**Table 8:** Illustrative quotes on Ukrainian farmers’ and veterinarians’ preparedness before 2022 and post-invasion support

[About preparedness for the war] We had no idea. And the situation is such that not even during the first week, or the second week, could one believe that something like this could happen. Sometimes you sit there, and even now it’s very hard to believe it. (Interview no 1)	
We had no plans. We were living our lives as usual. There were only a few hours left until the occupation. (Interview no 14)	
[About challenges related to state support] Currently, all government support is suspended until the war is over. There are programs where they are working on projects to help pig farming, but there are no active programs yet. (Interview no 12)	
[About challenges related to state support] The state probably shouldn’t support us too much. After all, there were others to support, right? I mean, there’s the defense forces and so on. So we support the army. So we don’t really expect much support from the state. And there never really has been either. (Interview no 8)	

### Key factors for farm resilience in crisis situations

All interviewees identified several critical factors for improving the resilience of livestock farms and animal-sourced food production during crises such as armed conflict. They consistently emphasised the importance of human resources, technical preparedness, and clear contingency planning. Skilled and well-prepared staff were seen as one of the most crucial assets, with training in crisis response and first aid for people and animals as well as clear roles and psychological readiness highlighted as essential. One farmer summarised this priority clearly, stating that “*the first and most important factor is the people*,* the staff*” (Interview no 1). One farmer stressed the urgent need to introduce first aid training for people on the farm, underlining that in times of crisis, the ability to act swiftly and provide proper assistance can be lifesaving, and should therefore be an integral part of future preparedness strategies.

All farmers except one identified reliable energy supply as a key factor, highlighting preparedness strategies such as access to multiple generators, sufficient fuel reserves, and alternative energy sources like solar panels as essential for sustaining operations. The availability of essential reserves, including feed, veterinary medicines, spare parts, and water, was also mentioned as critical to sustaining livestock production during disruptions. The farmers also stressed the importance of having reserves large enough to cover long time periods, one farmer mentioned at least 6 months to avoid immediate vulnerability.

Also, clear emergency plans were seen as necessary, especially those covering evacuation strategies for both animals and staff, alongside the flexibility to adapt operations as conditions change. Some farmers also underlined the importance of secure water supplies, adequate storage for products, and reliable transport solutions to ensure continuity of production and distribution. Furthermore, some farmers emphasised that establishing robust networks with suppliers, veterinarians, and neighbouring farms was essential for maintaining support and facilitating the exchange of critical resources during crises. In addition, the ability to adjust production and maintain sales channels, including on-farm processing, increased both autonomy and resilience. However, while many measures can mitigate risks, farmers also acknowledged that direct exposure to conflict zones may still lead to unavoidable losses. One farmer reflected on this stating: “*Even if there is feed available and whatever the circumstances*,* when the farm is surrounded and Russian troops are two kilometres away where fighting is going on*,* it does not matter if you have reserves. The farm could be bombed and destroyed at any time. It is important to make the right decisions to avoid suffering for the animals and to avoid risking the lives of the entire staff”* (Interview no 11). This underscores the need for flexibility and rapid decision-making in extreme situations. See Table 9 for more illustrative quotes.

**Table 9:** Illustrative quotes on key factors for farm resilience in crisis situations

The most important thing is, of course, the people […] Yes, basically how the team functions, all of that is crucial. (Interview no 1) The staff, yes, the most important thing is not to lose optimism. Humor helps us a lot. We have a very good team, we have such a team, and we are almost, well, the core team, so to speak, yes, it has hardly changed. (Interview no 3) That is to say, maybe the reserve should have been for two months, not three months. There’s no reason for me to buy more than that. Especially since it was unclear whether these supplies would be useful, whether there would be any evacuation, and that also makes it questionable. (Interview no 12) I wouldn’t work if I didn’t have a feed base for six months on the farm, fuel. Yes, in short, everything that ensures the farm’s operation, it must be there for the next six months. (Interview no 6) […] if it’s municipal water supply, maybe there’s no water. So, for example, if it’s about poultry facilities, there must be large tanks — ten, twenty, thirty tons, depending on the number of animals. We have tanks at many poultry facilities because anything can happen to the water, and birds don’t survive long without water. (Interview no 8)	

## Discussion

This study examines the challenges and lessons learned by some Ukrainian farmers and veterinarians following the Russian invasion in 2022. All interviews revealed the need for production adjustments, with key differences between occupied and non-occupied areas. These findings align with prior research showing that frontline and occupied regions in Ukraine faced the greatest economic losses in livestock production [17], and mirror evidence from Ethiopia, where conflict similarly reduced crop and livestock productivity [18].

Across interviews, there was a strong emphasis on safeguarding both personnel and animals, prioritizing welfare over profit. Despite psychological stress, displacement, and fatalities, respondents demonstrated resilience and a commitment to adaptation. Comparable patterns have been observed in Ghana, where livestock farmers persist despite drought and conflict, supporting both livelihoods and the national economy [19, 20].

For dairy farmers, occupation caused severe disruptions. Milking frequency had to be reduced due to feed shortages, staff losses, and animal stress, sharply lowering yields. Dairy farmers were cut off from processors and forced to give away, repurpose, or discard milk, while slaughterhouse closures created additional challenges such as overcrowding. Similar issues affected dairy supply chains in China and the United States during the COVID-19 pandemic [21]. Pig producers in occupied areas faced destruction and supply shortages, forcing them to release or donate animals, raising major animal welfare concerns. A major poultry farm also suffered catastrophic losses—challenges also reported in conflict-affected Ethiopian regions [22]. Operators outside of Russian-occupied territories reported varying degrees of production changes in response to the war, largely influenced by market disruptions, logistical constraints, and a focus on sustaining animal welfare and workforce stability. Some farms established alternative logistical channels and entered new markets, and a few even increased their production. Similarly, other research from Ukraine indicates that some companies operating outside conflict zones experienced a relative increase in revenues [23] and that many businesses quickly adapted to the new situation [24], although these studies do not focus specifically on farming and food production. Nevertheless, the underlying mechanisms—such as reorganising supply networks, diversifying markets, and strengthening crisis preparedness—remain relevant for livestock production.

Frequent and prolonged power cuts disrupted farm operations across all locations, affecting critical processes such as feed preparation, milking, water supply, and refrigeration. A recent report confirms that small- and medium-sized Ukrainian farmers continue to struggle with power outages, resulting in reduced production volumes [25].

Access to clean water varied but was sometimes compromised due to dependence on electric pumps. In conflict zones, infrastructure damage and attacks on pump stations forced reliance on unsafe sources like lake or rainwater. Restoring irrigation systems, including secondary networks, is reported to be vital for boosting agricultural production in areas impacted by the Kakhovka Dam’s destruction [26].

The conflict also caused severe disruptions to agricultural logistics including feed supply chains, transporting of live animals and products to markets, due to widespread infrastructure destruction and hostile territorial control. These challenges led to major economic losses, as supported by a recent report from Ukraine where logistical disruptions are emphasised as one of the primary challenges facing the agricultural sector [26].

Access to veterinary services, medicines and disinfectants was also disrupted, with negative impacts on animal health and welfare, although conditions gradually improved with external aid and resumed routines. Notably, no major rise in animal diseases on the farms was reported, contrasting with findings from Zimbabwe [8] and Afghanistan [9].

The war also caused severe workforce disruptions, resulting in acute labour shortages. Although staffing has partially stabilized, recruitment remains a significant issue. Labour shortages have also been highlighted as a key factor in reduced production in a recent report by the Ukraine Crisis Analysis Team [26].

One of the final questions addressed preparedness plans before 2022. It became clear that none of the farmers had anticipated war and were largely unprepared, lacking formal crisis plans. A similar failure to believe that a full-scale invasion would really happen, despite existing plans, is reflected in other research [24]. Crisis preparedness has even been referred to as ‘mission impossible’ due to the multi-faceted challenges [27]. Most farms relied on basic self-sufficiency, with limited stockpiles of fuel, feed, and medicine, and few emergency systems like generators or shelters. This shows the difficulty of planning for low-probability, high-impact events. As a result, improving preparedness will require not only individual effort but also external support, for example through targeted training programmes, subsidies for emergency equipment, and improved access to veterinary and logistical resources.

As previously discussed, the onset of conflict caused major disruptions, prompting improvised solutions and reliance on external support. These challenges have since motivated some farms to improve resilience and develop better emergency strategies. Similarly, research from Germany on the 2021 Western Europe floods found that, despite widespread risk awareness, actual preparedness remained low [28].

When asked to identify key strategies for enhancing livestock farm resilience during crises like armed conflict, respondents stressed the need for well-trained personnel, especially in preparedness planning, crisis management (including handling people under stress and quickly evacuating animals), and first aid for both humans and animals. Ensuring a stable energy supply through multiple generators and alternative sources was considered essential, along with long-term reserves of feed, water, and veterinary supplies. Respondents also emphasized clear, adaptable emergency plans, secure storage, reliable transport, and strong support networks with suppliers and neighbouring farms. Several of the proposed measures are achievable but depend on broader infrastructure functioning and stable supply chains. In active conflict settings, these conditions are often compromised, which limits what individual farms can realistically accomplish independently. While these measures help mitigate risks, farmers noted that proximity to active conflict zones can still result in unavoidable losses—highlighting the importance of flexibility and rapid, safety-focused decisions. This underscores that while the recommended measures can enhance resilience, they cannot eliminate risk. Thus, the most feasible strategies appear to be those that strengthen flexibility, rapid decision-making, and safety-oriented responses rather than those requiring extensive long-term investments that may be destroyed or inaccessible during conflict.

The study’s limitations include a small sample size, reducing generalizability. The interviews were not intended to be representative for Ukraine’s animal-sourced food production, but rather to capture some experiences and lessons learned during conflict. Despite the limited number of interviews, recurring themes across varied regions and respondents suggest a degree of consistency and reliability. Future research should expand to a wider range of regions and stakeholders across the food system, including suppliers, processors, distributors, authorities, and farmer associations. Their perspectives on supply chain disruptions and emergency response would help validate and deepen the insights gained from farmers and veterinarians.

## Conclusions

This study sheds light on the profound and multifaceted impact of the Russian invasion on key actors in Ukrainian livestock production, highlighting both the vulnerability and resilience of farmers and veterinarians operating under extreme conditions. The findings illustrate how proximity to active conflict zones shapes the scale and nature of disruption, with occupied areas experiencing the most severe breakdowns in production, logistics, and animal welfare. Key lessons from this study include the importance of maintaining long-term reserves and backup systems, the need for robust emergency protocols and support networks, and the essential role of skilled personnel. The results also highlight that even the best-prepared operations remain susceptible to the uncontrollable nature of war and a critical need for anticipatory resilience planning in regions vulnerable to geopolitical instability. The experiences of Ukrainian farmers and veterinarians provide important insights into how agricultural systems can become more adaptive and responsive during future crises, emphasizing the need for flexibility, preparedness, and community collaboration.

## Supplementary Information

Below is the link to the electronic supplementary material.

Supplementary Material 1.

## Data Availability

The datasets used and/or analysed during the current study are available from the corresponding author on reasonable request.
