# Supplementary material for: Maintaining animal-source food production in conflict zones: lessons from Ukraine
Source: Acta Vet Scand. 2026 Jan 7;68:4. doi: 10.1186/s13028-025-00850-5 (PMC12829245; doi:10.1186/s13028-025-00850-5)
Supplement: Supplementary file 1 — Supplementary Material 1. [file 13028_2025_850_MOESM1_ESM.pdf]

Additional file 1 – Interview guides

To the farmers

1. Region?

Ockupied or not?

Gender:

2. Type of production and number of livestock

- Pigs (sows, slaughter pigs)
- Dairy cows
- Beef cattle
- Broilers
- Hens for egg production
- Other type of livestock production – specify

3. Have you changed your production or work on your farm in any way since the war started in February 2022?

4. Could you describe the challenges you have encountered in your farming or animal food production activities since the onset of the war in February 2022?

5. Did you have any type of preparedness on your farm in case a crisis or conflict were to occur before 2022? If yes, can you describe what kind of preparedness you had?

6. Looking back at the past two years since the war started – can you name a few key factors on the farm that you think are especially important to be prepared for in the event of a crisis or conflict in order to maintain secure food production?

7. If you have any other comments connected to livestock production that you would like to share, we would be grateful to receive them.

To veterinarians

1. Region

2. Type of veterinarian?

Gender:

3. Have you changed your work as a veterinarian in any way since the war broke out in 2022? In terms of both the content of your work and the way you work?

4. Can you describe any challenges you have encountered in your work as a veterinarian since the war began (February 2022)—both in terms of treating sick animals and in preventive work, such as preventing outbreaks of infectious diseases?

5. Did you have any type of preparedness in place related to your work as a veterinarian in case a crisis or conflict were to occur before 2022? If yes, can you describe the kind of preparedness you had?

6. Looking back on the past two years since the war began – can you mention some key factors that you believe are especially important to be prepared for in the event of a crisis or conflict, in order to maintain safe production of animal-sourced food?

What is important for farmers to have in place as part of their preparedness?

What is important for veterinarians to have in place as part of their preparedness?

7. If you have any additional comments you would like to share, we would be grateful to receive them.
